# Enhancing IoT Network Security: Unveiling the Power of Self-Supervised Learning against DDoS Attacks

**DOI:** 10.3390/s23218701

**Published:** 2023-10-25

**Authors:** Josue Genaro Almaraz-Rivera, Jose Antonio Cantoral-Ceballos, Juan Felipe Botero

**Affiliations:** 1 Tecnologico de Monterrey, School of Engineering and Sciences, Monterrey 64849, Nuevo Leon, Mexico; a00821189@tec.mx; 2 Universidad de Antioquia, Electronics and Telecommunications Engineering Department, GITA-Lab, Medellin 050010, Antioquia, Colombia

**Keywords:** computer vision, contrastive learning, DDoS attacks, deep learning, Intrusion Detection System, IoT networks, self-supervised learning

## Abstract

The Internet of Things (IoT), projected to exceed 30 billion active device connections globally by 2025, presents an expansive attack surface. The frequent collection and dissemination of confidential data on these devices exposes them to significant security risks, including user information theft and denial-of-service attacks. This paper introduces a smart, network-based Intrusion Detection System (IDS) designed to protect IoT networks from distributed denial-of-service attacks. Our methodology involves generating synthetic images from flow-level traffic data of the Bot-IoT and the LATAM-DDoS-IoT datasets and conducting experiments within both supervised and self-supervised learning paradigms. Self-supervised learning is identified in the state of the art as a promising solution to replace the need for massive amounts of manually labeled data, as well as providing robust generalization. Our results showcase that self-supervised learning surpassed supervised learning in terms of classification performance for certain tests. Specifically, it exceeded the F1 score of supervised learning for attack detection by 4.83% and by 14.61% in accuracy for the multiclass task of protocol classification. Drawing from extensive ablation studies presented in our research, we recommend an optimal training framework for upcoming contrastive learning experiments that emphasize visual representations in the cybersecurity realm. This training approach has enabled us to highlight the broader applicability of self-supervised learning, which, in some instances, outperformed supervised learning transferability by over 5% in precision and nearly 1% in F1 score.

## 1. Introduction

The Internet of Things (IoT) encompasses a broad range of applications, spanning from smart homes to smart cities. It embodies the integration of physical objects, such as wireless healthcare devices, agricultural irrigation systems, and smart grid electric panels, with internet connectivity [1]. The global count of IoT connections is projected to exceed 30 billion by 2025 (IoT active device connections worldwide from 2010 to 2025. https://www.statista.com/statistics/1101442/iot-number-of-connected-devices-worldwide/, accessed on 10 July 2023), thereby amplifying the attack surface susceptible to security breaches. These breaches primarily include denial-of-service attacks (DoS and DDoS) [2], as well as unauthorized data extraction, given the frequent collection and exchange of confidential data by IoT devices [3].

Given the impulse that 5G networks [4] and Software-Defined Networking (SDN) [5] allow for IoT expansion [6], Artificial Intelligence (AI) has been used as a crucial tool for the development of Intrusion Detection (IDS) and Prevention Systems (IPS) [7,8]. These AI-empowered systems scrutinize traffic within a host or a network, trigger alerts, and counter potential threats in real time. However, achieving the anticipated high detection rates requires datasets that reflect contemporary attack scenarios and network traffic patterns.

Indeed, the scarcity of recent and robust data collections has been identified as a significant gap in contemporary research [9,10,11]. Given the heterogeneous and resource-constrained nature of IoT devices [12], popular datasets like CIC-IDS2017 [13] may not be apt for training necessary machine learning (ML) and deep learning (DL) models, mainly due to the lack of IoT devices in their testbeds. Consequently, alternative datasets have been proposed in the literature, including, but not limited to, Bot-IoT [14], TON_IoT [15], CIC IoT [16], and LATAM-DDoS-IoT [17].

Nonetheless, while the availability of the aforementioned datasets addresses the issue of suitable data quality for the IoT, the success of AI-based IDSs and IPSs is also contingent upon the chosen training strategy. Supervised learning necessitates copious amounts of labeled data to construct predictive models. In contrast, unsupervised learning does not require such ground truth information, but it presents challenges with generalization [18], specifically its limited ability to adapt to unseen, related data. This becomes especially relevant in the face of a rapidly evolving threat landscape, with new types of attacks emerging daily.

Self-supervised learning (S-SL) is a promising solution to challenges such as the demand for vast amounts of manually labeled data and the imperative for robust generalization [19]. In fact, S-SL is also considered suitable for dealing with the problems of small and imbalanced datasets [20]. This innovative approach bridges supervised and unsupervised learning. Initially, a model undergoes pre-training without labels, employing either auxiliary pretext tasks or contrastive learning, with the objective of capturing latent representations of the knowledge domain. Subsequently, this pre-trained model is fine-tuned using labeled data for specific downstream tasks, like attack detection or malware family classification [21]. Even though labeled information is still required for this later phase, Few-Shot Learning (FSL) [22] has demonstrated to be enough to obtain strong performance [23]. FSL targets obtaining strong learning performance given a limited number of labeled samples in the training set [22].

S-SL stands as a promising direction for ML advancements [24]. Today’s landscape features a plethora of models adept at leveraging this pioneering training methodology to extract insights from vast amounts of unlabeled data. Examples include Barlow Twins [25], SimCLR [26], Vision Transformers [27], Bootstrap Your Own Latent (BYOL) [28], and Momentum Contrast (MoCo) [29].

Contrastive learning, a training strategy for S-SL, aims to draw similar (or positive) examples closer while distancing dissimilar (or negative) examples [30]. This method capitalizes on data augmentation techniques to learn robust feature representations.

Therefore, in this manuscript, we create a smart IDS for detecting DDoS attacks against IoT networks using S-SL with the contrastive learning strategy [31]. Specifically, we compare the linear evaluation, i.e., the training of a fully connected layer on top of frozen representations [32], of a supervised pre-trained ResNet-34 architecture [33] with respect to its unsupervised counterpart using MoCo v2 [34]. Additionally, we detail the procedure for generating images from flow-level network traffic using the Bot-IoT and LATAM-DDoS-IoT datasets.

The conversion process from flow-level traffic into synthetic images was motivated by the success of contrastive learning of visual representations discussed in [35]. Furthermore, we decided to use the Bot-IoT and the LATAM-DDoS-IoT datasets since they provide attack traffic directed to virtual and physical IoT devices, as well as normal traffic information based on virtual machines and real users from a production network.

In summary, the primary contributions of this research are as follows:

- The pioneering of experimentation in IoT networks by leveraging the self-supervised learning paradigm in tandem with synthetic image generation, enabling the application of computer vision (CV) techniques for denial-of-service attack detection;
- The pre-training of self-supervised learning models using MoCo v2 on the Bot-IoT and the LATAM-DDoS-IoT datasets, laying the groundwork for fine-tuning in future specialized research tasks;
- An optimized training framework for future studies focusing on the contrastive learning of visual representations for the detection of denial-of-service attacks within IoT networks.

The remaining structure of this paper is divided as follows: the related work is presented in Section 2. In Section 3, we describe the process of creating the synthetic images, as well as the training of the ResNet-34 and MoCo v2 architectures. The results and discussion are detailed in Section 4, and the conclusion and future work are presented in Section 5.

## 2. Related Work

Here, we present related research on the creation of synthetic images for DDoS attack detection against IoT networks, as well as the learning strategies used to train the corresponding AI-based IDSs/IPSs.

While the existing literature reports various detection techniques, including those that analyze network traffic at the flow-level through recurrent models such as Recurrent Neural Networks, Long Short-Term Memory, and Gated Recurrent Units [36,37], our study specifically concentrates on pattern recognition through visual representations, which is a recognized research avenue that holds considerable potential for enhancing security measures in IoT environments [38,39,40].

In [41], the authors trained a ResNet-34 architecture in a supervised way using the CICDDoS2019 dataset [42]. This dataset was chosen since it includes 11 different types of denial-of-service attacks (e.g., SYN flood and UDP flood) described by 80 traffic features. To transform the flow-level traffic into images, the authors employed min-max normalization [43]. Each feature’s value was re-scaled between 0 and 1 and subsequently multiplied by 255. The resulting input images for the model measured 224 × 224 pixels and had three channels. For training, Stochastic Gradient Descent (SGD) [44] with a learning rate of 0.0001 and with a momentum of 0.9 was used. The model was trained for 10 epochs for binary classification and extended to 50 epochs for multiclass classification. The proposed solution achieved an accuracy of 99.99% and 87.06% for the binary and multiclass problems, respectively. Notably, while the authors devised an AI-based solution for denial-of-service attack detection in IoT networks, they neither tested their model in an environment with IoT devices nor sourced a dataset from IoT traffic. The CICDDoS2019 dataset they used originates from a testbed setup involving a victim web server and Windows PCs.

Reference [45] proposed an anomaly-based IDS using ResNet-50 with convolutional layers of one dimension. This system was trained using three different datasets, namely the NSL-KDD (NSL-KDD dataset. https://www.unb.ca/cic/datasets/nsl.html, accessed on 12 July 2023), CIC-IDS2017, and UNSW-NB15 [46], covering several categories of attacks, including denial-of-service, reconnaissance, and brute force. The input data were not transformed into images, but instead, the sequential traffic was fed into the model for classification purposes. The proposed smart IDS outperformed other AI models, such as Decision Trees, Random Forests, and Support Vector Machines, as in the case of UNSW-NB15, with a maximum accuracy of 92.18% and an F1 score of 89%. Nevertheless, the experiments conducted in the paper ignored the S-SL paradigm, as well as IoT traffic.

The authors of [18] created a network-based IDS based on S-SL and grayscale images obtained from preprocessing the UNSW-NB15 dataset. Contrastive learning was followed, with a data augmentation policy that included operations such as vertical flipping and random cropping. For the AI approach, the authors utilized the BYOL model, which consists of two neural networks (online and target) that learn from one another through data augmentation. The BoTNet [47] encoder was selected as the feature extractor, and the generalization ability of the proposed IDS was evaluated with fine-tuning on the NSL-KDD, KDD CUP 99 [48], CIC-IDS2017, and CIDDS_001 [49] datasets. Even though these S-SL experiments outperformed purely supervised learning models in some cases by more than 5% in terms of accuracy, this work was not tested on an IoT-related scenario.

In [50], a custom model based on BYOL was proposed, pre-trained using S-SL and contrastive learning on the UNSW-NB15 dataset. Regarding data augmentation, the authors applied masking, which consisted of randomly assigning a value of zero to a predefined percentage of features of each input sample. The transferability of the proposed model was evaluated under the Bot-IoT dataset, presenting an accuracy of 99.83% and an F1 score of 99.82%. Although there are experiments around the IoT domain, the pre-training phase used the UNSW-NB15 dataset, which may have negatively affected the feature representation quality of the model for IoT networks.

The authors of [51] used S-SL and contrastive learning along with the UNSW-NB15, CIC-IDS2017, and CSE-CIC-IDS2018 (CSE-CIC-IDS2018 dataset. https://registry.opendata.aws/cse-cic-ids2018, accessed on 13 July 2023) datasets for creating a network-based IDS using a custom model with a Multi-layer Perceptron (MLP) as the backbone. With respect to the data augmentation strategy, the authors generated adversarial examples based on [52]. The accuracy for DoS attack detection was 97.63% using the MLP model with the S-SL strategy, compared to the 54.34% accuracy of the MLP model without the S-SL pre-training process. Although these results reflect the potential of S-SL when compared to a purely supervised learning training strategy, the work presented in [51] might benefit from extending its experimentation to more testbeds, such as those of smart homes and industrial IoT environments.

Table 1 provides a comprehensive breakdown of the studies discussed in this section, analyzed across four distinct aspects. Specifically, the table examines if the proposed IDS was trained via the S-SL approach, the application of contrastive learning, and the use of IoT traffic during the pre-training phase. Based on this review, our manuscript distinguishes itself in the current literature. It emerges as the sole study implementing S-SL with contrastive learning to devise a network-based IDS, pre-trained using IoT traffic and tailored for detecting denial-of-service attacks.

## 3. Methodology

**Figure 1:**
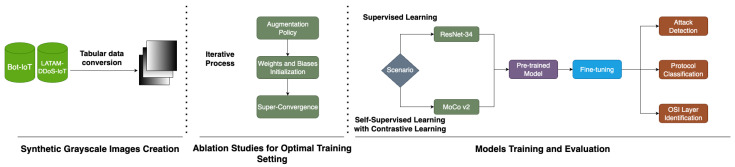
Methodology’s three main phases: the creation of synthetic images, the design of different ablation studies to find the optimal training setting, and the models’ training and evaluation.

This section presents the steps we followed to create our smart IDS. In Figure 1, there is a detailed breakdown of the three main phases that comprise this research, namely, the generation of synthetic grayscale images from sequential IoT traffic, then the design and implementation of different ablation studies to find the optimal training setting, and finally, the training and evaluation of models under the supervised learning and S-SL scenarios.

### 3.1. Synthetic Image Creation

The Bot-IoT and LATAM-DDoS-IoT datasets structure their input samples sequentially. We transformed this traffic into grayscale images to make the instances compatible with our 2D convolutional layers. This conversion involved applying min-max normalization and gamma correction, reshaping the input data, and then multiplying by 255 to represent various pixel intensities (as illustrated in Equation (1)). We utilized the second feature set proposed by [53], which comprises 15 statistical variables. These are detailed in Table 2 and encapsulate crucial data such as the bidirectional flow of packets and bytes exchanged between attackers and victims.

(1) $$x^{\prime }={\left(\frac{x-mean}{stddev}\right)}^{\gamma }\times 255$$

Gamma correction was employed with the aim of enhancing the magnitudes of features following the normalization process, thus preventing potential information loss [54,55]. In this process, all values ranging from 0 to 1 are rescaled. Through manual testing, an optimal gamma value of 0.1 was identified. As a result, a feature originally set at 0.0065 escalates to 0.6044, and another feature initially at 0.8295 is adjusted to 0.9815. This transformation not only bolsters the significance of each feature within the pixel grid but also maintains their relative ordering.

**Figure 2:**
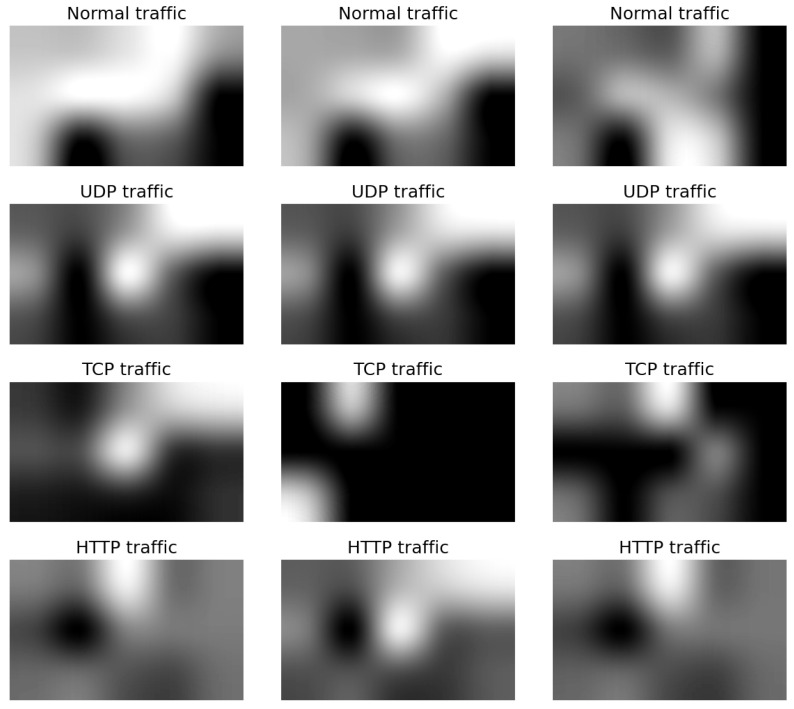
Synthetic grayscale images generated from the Bot-IoT dataset. The categories UDP, TCP, and HTTP represent the attack classes.

**Figure 3:**
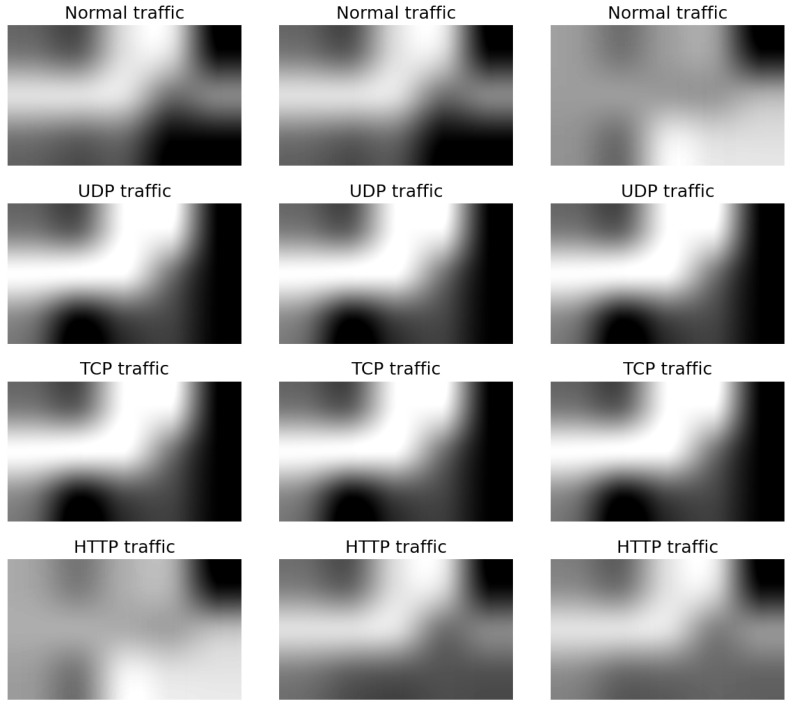
Synthetic grayscale images generated from the LATAM-DDoS-IoT dataset. The categories UDP, TCP, and HTTP represent the attack classes.

Figure 2 and Figure 3 show the grids of the different grayscale synthetic images generated from the Bot-IoT and the LATAM-DDoS-IoT datasets, respectively. We show three instances per traffic class for comparison purposes between the samples. Visually, intra-class similarities may be seen in terms of the location of black or white spots in small local regions in the images. The lower the magnitude of a feature translates into a darker spot, whereas the lighter pixels indicate higher feature values. The synthetic image for each input flow is of size 500 × 300.

Although converting sequential traffic into image form introduces an additional processing step in our classification tasks, the relevance of Convolutional Neural Networks in the state of the art has furthered the exploration of data transformation methodologies to leverage these architectures [39]. Furthermore, the graphical representation of the traffic aids for data visualization purposes.

### 3.2. Model Training and Evaluation

For this study, we tried two distinct data augmentation policies, both incorporating random cropping, Gaussian blur, and horizontal flipping, with the addition of random noise being the only difference between them. This noise was applied by multiplying each image pixel by a unique random value within the range of 0.8 to 1.2. This approach was inspired by [56], who found that training AI models on noisy data led to improved accuracy and robustness. We chose to implement MoCo v2 because it can process a large quantity of negative samples without requiring extensive training batches [34], enabling us to conduct training without TPU support.

**Figure 4:**
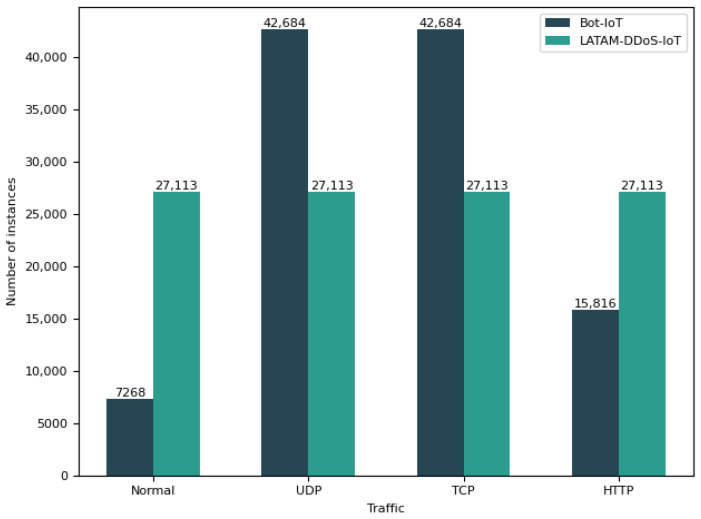
Data distribution for the pre-training phase.

The number of samples used for pre-training was 108,452, with a batch size of 128. Given the relatively small batch size, we did not implement MoCo v3 [57], an incremental advancement over MoCo v1 and MoCo v2, which have been observed to provide diminishing returns with larger batch sizes (e.g., 4096) [57]. To avoid any related bias due to the training data distribution, we took care of the ratios between classes. Hence, the 108,452 samples from the LATAM-DDoS-IoT are evenly split into 27,113 instances each for the normal, UDP, TCP, and HTTP classes. For the Bot-IoT, the UDP and TCP categories have 42,684 samples each, while the normal and HTTP classes have 7268 and 15,816 instances, respectively, since the Bot-IoT does not provide a sufficient number of flows for these two categories [14] (see Figure 4).

Additionally, we conducted an ablation study to assess the effect of initializing model weights and biases using ImageNet [58] and another study about applying cyclical learning rates [59]. The latter involved implementing a one-cycle learning rate policy for super-convergence [60].

Details regarding the pre-training process are as follows:

- The SGD optimizer was chosen for MoCo v2, while the Adam optimizer [61] was employed for the supervised learning approach.
- For experiments involving cyclical learning rates, the SGD optimizer was consistently used. Both learning strategies typically employed the cosine annealing learning rate scheduler, except during evaluations of the one-cycle learning rate policy.
- Batch normalization yielded means and standard deviations of 0.4367 and 0.2715, respectively, for the LATAM-DDoS-IoT dataset and 0.3414 and 0.2202 for the Bot-IoT dataset.

The details of the fine-tuning process are as follows:

- We used a batch size of 32, the Adam optimizer, and the cosine annealing learning rate scheduler.
- Overall, both the pre-training and fine-tuning phases spanned 100 epochs each.

ResNet-34 served as the backbone for both the supervised learning paradigm and MoCo v2 in all experiments. The models were designed using PyTorch [62] and were trained on a multi-GPU Ubuntu system equipped with two Tesla M10 accelerators.

The following metrics were chosen to measure the classification performance: accuracy, precision, recall, and F1 score. See Equations (2)–(5) for the definition of these metrics. For a binary classification problem such as attack detection, TP stands for true positives (i.e., the number of attacks classified correctly), TN refers to true negatives (i.e., the number of normal traffic samples classified accurately), FP indicates false positives (i.e., the number of normal traffic samples classified as attacks), and FN means false negatives (i.e., the number of attacks classified as normal traffic) [63].

(2) $$Accuracy=\frac{TP\;+\;TN}{TP\;+\;TN\;+\;FP\;+\;FN}$$

(3) $$Precision=\frac{TP}{TP\;+\;FP}$$

(4) $$Recall=\frac{TP}{TP\;+\;FN}$$

(5) $$F1\;score=2\times \frac{Precision\;\times \;Recall}{Precision\;+\;Recall}$$

### 3.3. Downstream Tasks Definition

We identified three specific downstream tasks:

- Attack detection: determining if an input image represents a DDoS attack;
- Protocol classification: classifying the input image based on its protocol (either UDP, TCP, HTTP, or standard traffic);
- OSI layer identification: recognizing the OSI layer the input image corresponds to, whether it is the transport layer, application layer, or standard traffic.

The multiclass protocol classification setup was also selected as the pre-training task for contrastive learning due to the complexity added by the number of classes (i.e., 4) compared to the other tasks (i.e., 3 classes for OSI layer identification and 2 for binary attack detection).

The proposed OSI layer identification task provides the groundwork to tackle security in a layered approach since each layer presents specific protocols and vulnerabilities. In practical terms, 5G is a potential scenario that benefits from this end-to-end protection, where each OSI layer must properly work to meet the design objectives of increased connectivity, lower latency, and high reliability [64].

For each downstream task, we employed only 300 labeled images per category. An exception was the OSI layer task, where the transport layer contained 600 samples split evenly between UDP and TCP images. Given the sparsely labeled data, these scenarios present a fitting examination of FSL capabilities for AI models.

It should be noted that the 108,452 instances utilized in the pre-training phase are intended to establish a robust feature representation for the problem of network intrusion within the IoT domain. The limited sample size in the fine-tuning phase is designed to assess the rapid adaptability of our models, leveraging prior knowledge to ensure both robustness and performance [65].

The results obtained from the different experiments are presented in the next section.

## 4. Experimental Results and Discussion

Here, we show the classification results from the supervised learning and S-SL scenarios and a corresponding discussion.

### 4.1. Ablation Studies to Find the Optimal Training Setting

This subsection uses the novel LATAM-DDoS-IoT dataset for the pre-training and fine-tuning phases to determine the optimal training setting from the ablation studies. These studies evaluated the augmentation policy for contrastive learning, the initialization of the models, and the concept of super-convergence.

See Table 3 for the linear classification results after evaluating the two augmentation policies discussed in the previous section. It can be seen that, in most cases, the augmentation policy without the noise operation leads to better classification performance (values highlighted in bold). This suggests that noise-free training data benefit both supervised learning and S-SL paradigms.

In our second experiment, we assessed the efficacy of employing pre-trained weights from ImageNet, as opposed to initializing the network randomly during the pre-training phase. As illustrated in Table 4, the S-SL model yielded significant performance enhancements compared to the supervised learning model. Specifically, we observed a 4.83% improvement in the F1 score for the task of attack detection and a 14.61% increase in accuracy for the multiclass protocol classification task. In contrast, as shown in Table 3, the supervised learning model mostly experienced marginal gains, often less than 1%, across various metrics. These results corroborate the notion that even when pre-trained on a dataset like ImageNet, which is unrelated to cybersecurity, the learned feature representations remain valuable for the synthetic image classification tasks in our study. This finding aligns well with existing literature, suggesting that the abstraction of image patterns, particularly in the initial layers of the network, is generally transferable across diverse visual domains [66,67,68].

The results in Table 5 show the linear classification performance obtained when employing an augmentation policy without the noise operation, utilizing ImageNet pre-trained weights, and implementing cyclical learning rates during the pre-training phase. Across all downstream tasks, we observed performance improvements for both supervised learning and S-SL paradigms, with certain tasks registering over a 5% increase in the F1 score. This demonstrates that, as stated in the original paper on super-convergence [60], the gains of the one-cycle learning rate policy shine when there are limited labeled training data available, as in the fine-tuning phases of this work.

### 4.2. Evaluating the Optimal Training Setting

Lastly, we implemented the optimal training setting under new testing scenarios, namely, an entire classification performance evaluation using the Bot-IoT dataset and transferability experiments having the LATAM-DDoS-IoT as the target.

See Table 6 for the linear classification results after using the Bot-IoT dataset for both the pre-training and fine-tuning phases. It may be seen that even though supervised learning outperforms S-SL, the difference tends to be minimal in all four metrics, in some tests as low as 0.02%.

Generalization, understood as the ability to predict unseen related data correctly, is a potential benefit of S-SL models [69]. Therefore, in Table 7, we present the linear results after pre-training the ResNet-34 and MoCo v2 models using the Bot-IoT dataset for the target LATAM-DDoS-IoT. This target dataset was used later for fine-tuning, and it may be seen that, in most cases, the S-SL approach outperformed the purely supervised learning scenario in some tests by over 5%. These values indicate that the feature representation learned by MoCo v2 is more robust than the one produced by the ResNet-34 architecture alone.

The modest gains in generalization attributed to S-SL may seem incongruent with the potential of this emerging training paradigm. One possible explanation for this discrepancy could be the selected pre-training task of multiclass protocol classification within the framework of contrastive learning. Identifying a sufficiently challenging pre-training task is often considered the most intricate aspect of effective S-SL, as it governs the quality of the learned feature representations [70]. To potentially enhance the detection rates of our models, an exploration of pretext tasks, such as the auxiliary task involving the shading and completion of segments within input images, may offer a promising avenue [71].

All the results presented in this section are the average of five runs, which indicates stable detection rates, and a benchmark where S-SL shows a competitive classification performance when compared to supervised learning.

### 4.3. Comparison with Previous Works

To the best of our knowledge, no existing study directly parallels our work. As discussed in Section 2, most related research primarily revolves around testbeds with web servers and PCs. While there is a study that experiments with the Bot-IoT dataset, the scope of [50] extends to multiclass classification, capturing a broader array of attacks, such as information theft [72], beyond just DDoS. Consequently, their findings are not directly comparable to our detection rates.

In the next section, we provide a conclusion for this study and outline potential avenues for future research.

## 5. Conclusions and Future Work

In this study, we showcased the efficacy of S-SL, positioning it as a suitable alternative to supervised learning in the context of linear classification performance. Leveraging two cutting-edge IoT network datasets, we introduced an innovative downstream task for classifying attacks via the OSI layer. Through comprehensive ablation studies, we outlined an optimized training setting emphasizing an augmentation policy devoid of random noise, with ImageNet initialization and a one-cycle learning rate scheduler. S-SL’s superior generalization aptitude was evident, outclassing supervised learning by over 5% in precision and nearly 1% in F1 score in certain tests.

Our results highlight the promise of S-SL in bolstering the security of IoT networks, thereby laying a foundational groundwork for future research endeavors and technological advancements. While we recognize an inherent limitation in our proposed solution, particularly concerning the computational overhead associated with converting each network flow into an image, our study nonetheless demonstrates the viability of leveraging S-SL to construct robust cybersecurity frameworks.

Looking ahead, we aim to harness S-SL for sequential data, possibly tapping into the capabilities of Transformers [73]. In addition, we will train with other datasets to capture even further diverse network traffic patterns [74], and we will experiment with a pretext tasks approach alongside other S-SL models for intrusion detection. Moreover, we plan to test these models under more realistic conditions, such as those from production networks with streaming data, to properly measure the flows/second (as a time performance metric [53]) each method may classify. Such studies will allow for a more comprehensive comparison against contrastive learning and aid in determining the most suitable S-SL strategy to achieve high detection rates in the cybersecurity domain.

## Figures and Tables

**Table 1:** Comparison between our work and the related state of the art around network intrusion detection for IoT.

	DoS/DDoS Attacks Traffic	Self-Supervised Learning	Contrastive Learning	IoT Pre-Training
Hussain et al. [41]	✔	✗	✗	✗
Shaikh and Gupta [45]	✔	✗	✗	✗
Wang et al. [18]	✔	✔	✔	✗
Lotfi et al. [50]	✔	✔	✔	✗
Deng et al. [51]	✔	✔	✔	✗
Our work	✔	✔	✔	✔

**Table 2:** Description of the 15 features used from the Bot-IoT and the LATAM-DDoS-IoT datasets. Table from [17].

Feature	Description
TotPkts	Total number of packets in the transaction.
TotBytes	Total number of bytes in the transaction.
Dur	Record total duration.
Mean	Average duration at records aggregate level.
StdDev	Standard deviation of the duration at records aggregate level.
Sum	Total duration at records aggregate level.
Min	Minimum duration at records aggregate level.
Max	Maximum duration at records aggregate level.
SrcPkts	Source to destination packets count.
DstPkts	Destination to source packets count.
SrcBytes	Source to destination bytes count.
DstBytes	Destination to source bytes count.
Rate	Total packets per second in the transaction.
SrcRate	Source to destination packets per second.
DstRate	Destination to source packets per second.

**Table 3:** Classification results: ablation study of the augmentation policy.

Downstream Task	Learning Paradigm	Augmentation Policy	Accuracy	Precision	Recall	F1 Score
Attack detection	Supervised Learning	with noise	85.29%	82.61%	52.12%	61.97%
		w/o noise	84.90%	79.69%	**52.55%**	61.52%
	S-SL	with noise	84.53%	78.88%	51.51%	60.36%
		w/o noise	**86.11%**	**81.38%**	**56.96%**	**65.26%**
Protocol classification	Supervised Learning	with noise	65.26%	70.81%	65.26%	64.12%
		w/o noise	**65.69%**	70.79%	**65.69%**	**64.79%**
	S-SL	with noise	55.39%	59.56%	55.39%	55.11%
		w/o noise	**75.85%**	**79.42%**	**75.85%**	**75.41%**
OSI layer identification	Supervised Learning	with noise	86.18%	89.73%	86.18%	85.26%
		w/o noise	86.06%	**89.88%**	86.06%	85.1%
	S-SL	with noise	79.59%	80.42%	79.59%	78.36%
		w/o noise	**86.29%**	**88.58%**	**86.29%**	**85.91%**

**Table 4:** Classification results: best augmentation policy and weights and biases initialization using ImageNet. The green values are the corresponding increases with respect to Table 3.

Downstream Task	Learning Paradigm	Accuracy	Precision	Recall	F1 Score
Attack detection	Supervised Learning	85.47% ↑0.57%	84.67% ↑4.98%	50.12%	61.02%
	S-SL	85.66%	77.85%	59.79% ↑2.83%	65.85% ↑0.59%
Protocol classification	Supervised Learning	64.81%	70.62%	64.81%	63.79%
	S-SL	79.42% ↑3.57%	81.85% ↑2.43%	79.42% ↑3.57%	79.23% ↑3.82%
OSI layer identification	Supervised Learning	86.21% ↑0.15%	90.4% ↑0.52%	86.21% ↑0.15%	85.14% ↑0.04%
	S-SL	85.24%	86.82%	85.24%	85.11%

**Table 5:** Classification results: one-cycle learning rate scheduler with the best augmentation policy and weights and biases initialization using ImageNet. The green values are the corresponding increases with respect to Table 4.

Downstream Task	Learning Paradigm	Accuracy	Precision	Recall	F1 Score
Attack detection	Supervised Learning	86.47% ↑1.0%	81.47%	59.26% ↑9.14%	66.81% ↑5.79%
	S-SL	85.99% ↑0.33%	80.92% ↑3.07%	56.8%	64.99%
Protocol classification	Supervised Learning	82.23% ↑17.42%	85.02% ↑14.40%	82.23% ↑17.42%	81.84% ↑18.05%
	S-SL	80.55% ↑1.13%	82.61% ↑0.76%	80.55% ↑1.13%	80.44% ↑1.21%
OSI layer identification	Supervised Learning	86.86% ↑0.65%	88.83%	86.86% ↑0.65%	86.55% ↑1.41%
	S-SL	85.31% ↑0.07%	86.85% ↑0.03%	85.31% ↑0.07%	85.22% ↑0.11%

**Table 6:** Classification results: pre-training and fine-tuning phases using the Bot-IoT dataset.

Downstream Task	Learning Paradigm	Accuracy	Precision	Recall	F1 Score
Attack detection	Supervised Learning	99.98%	87.51%	87.32%	87.39%
	S-SL	99.96% ↓0.02%	86.24% ↓1.27%	86.11% ↓1.21%	86.14% ↓1.25%
Protocol classification	Supervised Learning	99.90%	99.90%	99.90%	99.90%
	S-SL	99.85% ↓0.05%	99.86% ↓0.04%	99.85% ↓0.05%	99.84% ↓0.06%
OSI layer identification	Supervised Learning	99.97%	99.97%	99.97%	99.97%
	S-SL	99.95% ↓0.02%	99.95% ↓0.02%	99.95% ↓0.02%	99.95% ↓0.02%

**Table 7:** Classification results: generalization experiments using the Bot-IoT as the source dataset and the LATAM-DDoS-IoT as the target dataset.

Downstream Task	Learning Paradigm	Accuracy	Precision	Recall	F1 Score
Attack detection	Supervised Learning	84.95%	78.51%	54.87%	62.64%
	S-SL	85.45% ↑0.50%	84.18% ↑5.67%	51.85%	62.23%
Protocol classification	Supervised Learning	66.31%	70.26%	66.31%	65.85%
	S-SL	67.39% ↑1.08%	71.51% ↑1.25%	67.39% ↑1.08%	66.77% ↑0.92%
OSI layer identification	Supervised Learning	85.55%	87.92%	85.55%	85.06%
	S-SL	85.41%	88.02% ↑0.10%	85.41%	84.74%

## Data Availability

Two publicly available datasets were analyzed in this study. The Bot-IoT dataset can be found at https://research.unsw.edu.au/projects/bot-iot-dataset (accessed on 6 March 2023), and the LATAM-DDoS-IoT dataset at https://dx.doi.org/10.21227/rwtj-dd43 (accessed on 6 March 2023).
